# Exploring the relationships between psychological variables and loot box engagement, part 1: pre-registered hypotheses

**DOI:** 10.1098/rsos.231045

**Published:** 2023-12-20

**Authors:** James Close, Stuart Gordon Spicer, Laura Louise Nicklin, Maria Uther, Ben Whalley, Chris Fullwood, Jonathan Parke, Joanne Lloyd, Helen Lloyd

**Affiliations:** ^1^ Peninsula Medical School, Faculty of Health, University of Plymouth, Plymouth PL4 8AA, UK; ^2^ School of Psychology, University of Plymouth, Plymouth PL4 8AA, UK; ^3^ Community and Primary Care Research Group (CPCRG), ITTC Building, Davy Road, Plymouth Science Park, Derriford, Plymouth PL6 8BX, UK; ^4^ School of Education, Faculty of Education, Health and Wellbeing, University of Wolverhampton, Wolverhampton WS1 3BD, UK; ^5^ Enterprise and Innovation, Faculty of Health, Education and Life Sciences, Birmingham City University, Seacole Building, Edgbaston Campus, Birmingham, UK; ^6^ School of Natural, Sport and Social Sciences, University of Gloucestershire, Cheltenham, UK; ^7^ Sophro Ltd, Newark Beacon, Newark, UK; ^8^ Cyberpsychology Research Group, Department of Psychology, Faculty of Education, Health and Wellbeing, University of Wolverhampton, Wolverhampton WV1 1LY, UK

**Keywords:** loot boxes, video gaming, gambling, digital harms, addictive behaviours, wellbeing

## Abstract

Loot boxes are purchasable randomized rewards in video games that share structural and psychological similarities with gambling. Systematic review evidence has established reproducible associations between loot box purchasing and both problem gambling and problem video gaming, perhaps driven by a range of overlapping psychological processes (e.g. impulsivity, gambling-related cognitions, etc.) It has also been argued that loot box engagement may have negative influences on player financial and psychological wellbeing. We conducted a pre-registered survey of 1495 loot box purchasing gamers (LB cohort) and 1223 gamers who purchase other, non-randomized game content (nLB cohort). Our survey confirms 15 of our 23 pre-registered hypotheses against our primary outcome (risky loot box engagement), establishing associations with problem gambling, problem gaming, impulsivity, gambling cognitions, experiences of game-related ‘flow’ and specific ‘distraction and compulsion’ motivations for purchase. Results with hypotheses concerning potential harms established that risky loot box engagement was negatively correlated with wellbeing and positively correlated with distress. Overall, results indicate that any risks from loot boxes are liable to disproportionately affect various ‘at risk’ cohorts (e.g. those experiencing problem gambling or video gaming), thereby reiterating calls for policy action on loot boxes.

## 1. Introduction

Loot boxes are purchasable randomized content available in video games. We define loot boxes as characterized by: (i) being available for real-world money (even when ‘free’ offers are also available, or financed via the purchase of virtual/in-game currencies) and (ii) having randomized outcomes, where the digital contents (which might, for example, offer gameplay advantages or cosmetic upgrades) have varying financial or psychological value [1,2]. While other definitions of loot boxes exist [3], whatever definitions are used, loot boxes are nonetheless available in the majority of games on various platforms including console games, pc games and mobile games and can often be accessed by children [1,4].

With structural and psychological similarities to gambling [5], systematic review evidence of loot boxes has established robust associations with both problem gambling and problem video gaming [6–10]. While the motivations for purchasing are complex [11], it is known that high spenders on loot boxes (i.e. more than $100 per month, or local equivalent) are disproportionately represented by problem gamblers, but not higher earners [12]. This suggests that game developers are deriving outsized profits from at-risk individuals—likely to include problem gamblers, problem gamers and other at-risk cohorts—rather than simply from higher earners.

There has been much commentary that potential risks posed by loot boxes may disproportionately impact specific demographics, especially younger people [5,13]; males [14–16]; and those who are prone to greater impulsivity [17,18] or problem gambling behaviours [19–21]. Nascent evidence suggests such concerns may be warranted, and it has been established that links between problem gambling and loot box purchasing are stronger in adolescents [17]. Furthermore, our previous survey of around 16 000 gamers established associations between loot box engagement and a range of demographic variables, including younger age, male sex and non-university educational attainment and employment status [22].

In addition to such demographic variables, a range of psychological drivers may also be linked with loot box engagement. Impulsivity is a known psychological risk factor for disordered gambling [23]; and it has been previously suggested as a factor in loot box engagement [24]. Previous surveys, however, have presented equivocal evidence for links between impulsivity and loot box expenditure [17,18,25–27].

Similar to impulsivity, distorted cognitions about gambling—such as the illusion of control—are robustly associated with problematic gambling [28], and such cognitions have also been suggested as a factor in loot box engagement (albeit only in a single survey [29]).

There may also be direct game-related motivations that influence loot box engagement. To investigate such motivations, we previously conducted a qualitative investigation of the reasons and facilitators for videogame loot box engagement, categorizing them into a series of over-arching ‘themes’, such as social influences, the excitement of the opening experience and the ‘fear of missing out’ on opportunities to gain items [11]. Building on this qualitative research, we developed a 23-item scale for measuring these motivations, named ‘Reasons and Facilitators for Loot box Engagement’ (RAFFLE) [30].

It would be obvious to hypothesize that greater motivation on the RAFFLE scale would correlate with greater loot box engagement. However, the RAFFLE is divided into seven sub-scales, mapping approximately to the themes generated from our qualitative work: Enhancement; Progression; Social Pressure; Distraction/Compulsion; Altruism; Fear of Missing Out; Resale Value [30]. Here, the Distraction/Compulsion sub-scale contains items comprising aspects such as escapism, which is associated with disordered gaming [31] and gambling [32], and items encompassing ‘urges’, which are associated with problematic gambling. Therefore, similar to problem gambling and problem gaming, motivations of Distraction/Compulsion may be correlated particularly strongly with problematic types of loot box engagement.

In addition to outstanding questions around the psychological drivers of loot box engagement, academic commentators have also stressed the potential impacts of loot box engagement on player financial and psychological wellbeing [12,16,33,34]. While the detrimental effects of problem gambling are well established [35], only a limited number of studies have specifically investigated how engagement with loot boxes might influence (or be influenced by) player wellbeing. While one survey established that loot box purchasers had higher levels of mental distress [33], a second survey found no associations with loot box spending and either distress or mental wellbeing [36]. Interestingly, a third survey established that both positive *and* negative moods might be associated with greater loot box expenditure [37].

Furthermore, there are other previously unstudied psychological factors that also have the potential to drive loot box engagement, such as the concept of ‘flow’. Flow has been described as an experience of full immersion, where the challenges of an activity are well balanced with the skills of the person undertaking the activity [38,39], and the design of video games is fundamentally underpinned by such concepts. Within the field of gambling research, links have been established between experiences of ‘dark’ flow (a flow-like state of high absorption) and problematic slot machine play [40]. Here, the flow-like experiences of slot machines offer an escape motivation from mental health issues such as depression [40,41]. Despite this, no studies have investigated if such flow experiences—which have been linked with problematic gaming [42]—relate to loot box engagement.

### 1.1. The present study

Based upon the above literature, we designed a pre-registered survey [43] to capture how *all* these various gaming, gambling, demographic and psychological variables relate with loot box engagement. This study investigated the relationship of both randomized game-related purchases (i.e. loot boxes) and non-randomized game-related content (e.g. add-ons brought outright, like skins, weapons, downloadable content) to variables including problem gambling, problem gaming and wellbeing/distress in a UK cohort. Our primary outcome variable was not loot box expenditure, but instead the ‘risky loot box index’ (RLI). This has been used in several previous studies, shown to have significant relationships with both loot box purchasing and problematic gambling, and may represent a more direct measure of ‘risky’ or problematic engagement with loot boxes [29,37,44–46].

We pre-registered our hypotheses in three broad categories: (a) predictions of significant correlations between loot box engagement and a range of psychological instruments (measuring problem gambling, problem gaming, impulsivity, gambling cognitions and flow) and demographic variables (e.g. age, sex); (b) predictions that many of these correlations would be weaker with non-randomized (i.e. not loot box) game-related purchases; and (c) hypotheses around the putative psychological and financial harm associated with loot box engagement (such as associations between higher psychological distress and lower wellbeing). These hypotheses are detailed fully in the pre-registration document on the Open Science Foundation [43], and are summarized in the first five rows of table 2, providing the variables/instruments involved, the predicted direction and statistical benchmarks.

Additionally, the pre-registration document specified a number of exploratory regression analyses (i.e. where the methods were pre-registered, but we made no hypotheses about the actual results). Here, the extant literature on the psychology of loot box engagement has primarily relied on bivariate analyses [6–10]—i.e. similar to the pre-registered hypotheses presented here. Any relationships between psychological variables and loot box engagement were typically investigated on a one-by-one basis. However, the relationships between these various overlapping constructs (i.e. impulsivity, problematic gaming, problematic gambling, gambling cognitions, etc.) are likely to be complex and multidirectional [46]. Therefore, to gain new insights into the complex relationship between these variables—including which variables are most predictive of loot box engagement—we pre-specified a number of Bayesian mixed-effects regressions. Given the extensive nature of these exploratory regression analyses, they are dealt with separately in a second, linked publication: ‘The psychology of loot box engagement, part 2: exploratory analyses of complex relationships’*.*

## 2. Methods

Our study design was pre-registered on the Open Science Foundation [43]. The data and R code for running the analysis are openly available at https://osf.io/gh634/. Our approach targeted a UK cohort that included comparative data from a sample of both loot box and non-loot box purchasing gamers. Both cohorts responded to an extensive battery of measurement instruments producing a detailed profile of gaming, gambling, demographic and psychological variables.

### 2.1. Data collection and participant exclusion

Participants were recruited from the survey recruitment platform Prolific [47], resided in the UK, were adults (18+), and had been previously identified (via a short screening survey [22]) as videogame players who purchased game-related content. As specified in our pre-registration document, we sought to recruit a cohort of 1200–1500 participants who purchase loot boxes (loot boxers or LB cohort) and 1200–1500 participants who purchase only non-randomized game-related content (non-loot boxers or nLB cohort). Data were collected from both cohorts during a matched data collection window (8 March 2021 to 24 March 2021), on the Qualtrics [48] survey platform. All ordinal/categorical and free-text responses used ‘force response’ options, so that issues of missing data were avoided.

Participants were excluded if they failed one of two survey-based attention checks (e.g. ‘please select 3 to this question’ answered incorrectly). We also inspected data for duplicate IP addresses, possible ‘bots’ (from the automated Qualtrics ReCAPTCHA algorithm/scoring) and other non-serious answers, to ensure any suspicious responses were removed.

### 2.2. Survey questions and measurement instruments

Our hypotheses, described later, were predicated on measurements of loot box engagement, gaming, gambling, demographic variables, a variety of psychological instruments and measures of wellbeing and distress. These are described below and summarized in box 1.

Box 1. **Measurement instruments included in the survey. Within the text, these are usually referred to by the ‘concept’, rather than the instrument or abbreviated instrument name (abbr.)**

**Table:** 

concept	abbr.	instrument and reference
risky loot box engagement	RLI	Risky Loot Box Index [29]
problem gambling	PGSI	Problem Gambling Severity Index [49]
problem video gaming	IGD	Internet Gaming Disorder Short Form [50]
LB motivations	RAFFLE	Reasons and Facilitators for Loot Box Engagement Scale [30]
LB motivations sub-scales	RAFFLE Enhancement	
	RAFFLE Progression	
	RAFFLE Social	
	RAFFLE Distraction/Compulsion	
	RAFFLE Altruism	
	RAFFLE FOMO (Fear of Missing Out)	
	RAFFLE Resale	
gambling cognitions	GRCS	Gambling Related Cognition Scale [51]
impulsivity	BIS-Brief	Barratt Impulsiveness Scale (BIS-Brief) [52], eight-item version [53]
flow	GES-Flow	previously used five-item set of the Game Experience Questionnaire [40,41]
wellbeing	WEMWBS	Warwick–Edinburgh Mental Well-being Scale (WEMWBS) [54]
distress	K-10	Kessler Psychological Distress (K-10) [55]

#### 2.2.1. Loot box engagement

‘Loot box engagement’ has been variably defined in previous literature. The most frequently used standard has been typical or previous monthly spend. Alternatively, the RLI, a tool which measures risky or problematic engagement with loot boxes, has also been used in several studies [29,37,44,45]. While expenditure is a self-report measure of financial costs, the RLI is a conceptualization of ‘over-involvement’, capturing how people feel about their engagement—i.e. by asking them to rate their agreement with statements like ‘once I open a loot box, I often feel compelled to open another’. Therefore, our ‘primary outcome’ is the RLI, but we hypothesized that our pre-registered hypotheses would be robust to all common definitions of loot box engagement, namely: (i) typical monthly spend, (ii) last monthly spend and (iii) RLI.

It is also worth noting that there have been suggestions that previous observations of links between gambling and loot box engagement may be driven by the fact that players are confusing loot boxes as representing genuine gambling [56]. Our survey wording, therefore, made clear distinctions between loot boxes versus genuine forms of gambling, by (for example) first defining loot boxes as ‘chance-based videogame items'.

#### 2.2.2. Gaming/gambling related variables

We asked respondents about their typical, and most recent, monthly spend on loot boxes *or* non-loot box spending (i.e. depending on cohort).

#### 2.2.3. Demographic variables

The following demographic variables were automatically captured from Prolific: age (as a continuous variable), sex, employment status and ethnicity (Simplified on Prolific). We also asked respondents about gender and self-report earnings/salary.

#### 2.2.4. Measurement instruments

Our study included a range of previously validated measurement instruments (box 1). For all variables, we analysed continuous scores rather than any pre-existing categories based on putative diagnostic cut-off points (i.e. like those for PGSI and IGD for example). In addition to maximizing the sensitivity of the analyses, we were also interested in relationships across the full continuum of scores, rather than solely differentiating between groups based off arbitrary cut-off points. Recent analysis of risk curves of the PGSI provides support for this approach [57].

#### 2.2.5. Hypotheses

A brief overview is provided in table 2 of the results section (columns 1–5); summarizing each hypothesis, variables included, predicted direction and statistical benchmarks. With Hypothesis 1.2, we made a necessary (but inconsequential) deviation from the pre-registration document: here, we did not test whether RAFFLE would have an interaction with type of spend (where we predicted that the relationships will be stronger with loot boxes than non-randomized content). The final version of the RAFFLE [30] was designed to only measure loot box (rather than generic) game-related spend, and could, therefore, not be deployed to test this hypothesis.

#### 2.2.6. Statistical tests

We analysed all data in R [58]. For instrument scoring, we used standard, published methods of calculation, although we rescaled all responses from to zero [59] for consistency (which will not influence results). For hypotheses tested with bivariate correlations (table 2), we used both frequentist (or ‘null hypothesis significance testing’, NHST) and Bayesian approaches. Here, the majority of previous literature on loot boxes [12,60] has used non-parametric frequentist tests, reporting significance and effect sizes of bivariate correlations with loot box engagement. For consistency with this previous literature, we report bivariate correlations for associations specified in each hypothesis, using Kendall's tau for frequentist testing, and reporting *p-*values after applying false discovery rate correction (which corrects for multiple tests) to this set of bivariate tests [61]. An alpha level of 0.05 (after correction) was used as the threshold for significance. Additional to previous literature, we conducted Bayesian Kendall's tau tests, reporting Bayes factors and (where necessary) 95% credible intervals (CIs). In keeping with accepted practice [62], Bayes factors of more than three are interpreted as evidence of an effect, while a value less than one third is interpreted as evidence of no effect. Values between one third and three are interpreted as inconclusive. Any conflicting results between frequentist and Bayesian results are interpreted as a sensitivity analysis; any discrepancies are noted and discussed in the results section. For testing the binary variable of sex, we conducted frequentist Wilcoxon rank-sum test and Bayesian *t*-test; for the categorical variable of employment status we used frequentist Kruskal–Wallis test and Bayesian ANOVA.

For hypotheses that compared correlations between two tests (e.g. H1.2a, which predicts that PGSI will be more strongly associated with loot box engagement, compared with engagement with non-randomized purchases), we assessed Bayesian CIs; where our 95% CIs were not expected to overlap (providing evidence of a difference).

Finally, a multiple linear regression was conducted to test for an interaction between PGSI and IGD as predictors of loot box engagement (Hypothesis 2.5); where we predicted independent contributions from PGSI and IGD towards loot box engagement (i.e. no interaction in multiple regression).

## 3. Results

### 3.1. Survey responses, exclusions and final cohort

We received a total of 1551 responses for the LB cohort, and 1512 responses for the nLB cohort. We removed 71 participants (38 LB; 33 nLB) who failed one or both attention checks; 54 participants (19 LB; 35 nLB) who either did not complete all questions, or who revoked consent, or both; and 220 participants in the nLB cohort who stated they purchased loot boxes (note that these respondents had previously reported (in our pre-screen survey, see Methods) that they did not purchase loot boxes; they were removed because their loot box spend/engagement data were, therefore, incomplete). This produced a final cohort of 1495 for the LB cohort and 1223 for the nLB cohort. Anonymized, open access data for the finalized cohort are available on the Open Science Foundation (https://osf.io/gh634/).

See table 1 for the demographic characteristics of the two cohorts.

**Table 1.** Demographic composition of LB versus nLB cohort.

	loot box (LB)	non-loot box (nLB)
	*n* (%)	*n* (%)
*n*	1495	1223
gender		
M	711 (51.51%)	695 (56.83%)
F	770 (47.56%)	503 (41.13%)
non-binary/third gender	14 (0.94%)	17 (1.39%)
other/prefer not to say		8 (0.65%)
mean age (s.d.)	31.16 (9.93)	32.84 (10.53%)
ethnicity		
White	1253 (83.81%)	1042 (85.20%)
Asian	97 (6.49%)	57 (4.66%)
Black	40 (2.68%)	29 (2.37%)
mixed	70 (4.68%)	54 (4.42%)
other	9 (0.60%)	14 (1.15%)
unknown	26 (1.74%)	27 (2.21%)

### 3.2. Hypothesis testing

Table 2 summarizes our three broad categories of pre-registered hypotheses: (a) correlations between loot box engagement and demographic or psychological variables; (b) attenuated correlations with non-randomized (as compared to randomized) in-game purchases and (c) putative harms from loot box engagement. See box 1 for details about the instruments and table 2 for the hypotheses.

**Table 2.** Overall results of hypothesis tests. Lists the hypotheses, predictor/outcome variables of each hypothesis, predicted relationship between the variables and the type of statistical test conducted. For statistical tests, ‘NHST/Bayes’ = tested using both frequentist and Bayesian tests; ‘Bayes CI’ = test involves establishing that one association is stronger than the other, assessed using manual inspection of confidence intervals. The penultimate column lists whether the hypotheses are supported or not (‘+’ = hypotheses supported; ‘−’ = hypothesis not supported; ‘?’ = inconsistent results, which also lists the outcome variable that was supported). The column ‘full results’ provides a reference to the section of the paper that provides the full results.

hypothesis	predictor	outcome	predicted relationship	test	result	full results	possible reason for non-supported hypothesis or inconsistent result
*H1.1.1–H.1.1.8:*	*(a) bivariate correlations with psychological instruments and demographic variables*						
H1.1.1	problem gambling (PGSI)	loot box engagement (RLI/spend)	positive association	NHST/Bayes	+	table 3	
H1.1.2	problem gaming (IGD)	loot box engagement (RLI/spend)	positive association	NHST/Bayes	+	table 3	
H1.1.3	loot box motivations (RAFFLE)	loot box engagement (RLI/spend)	positive association	NHST/Bayes	+	table 3	
H1.1.4	loot box motivations (RAFFLE): Distraction/Compulsion sub-scale	loot box engagement (RLI/spend)	strongest association	Bayes CI	+	table 4	
H1.1.5	gambling cognitions (GRCS)	loot box engagement (RLI/spend)	positive association	NHST/Bayes	+	table 3	
H1.1.6	impulsivity (BISB)	loot box engagement (RLI/spend)	positive association	NHST/Bayes	?RLI only	table 3	impulsivity appears to have a specific link with risky loot box engagement
H1.1.7	flow (GES-flow)	loot box engagement (RLI/spend)	positive association	NHST/Bayes	+	table 3	
H1.1.8a	male sex	loot box engagement (RLI/spend)	positive association	NHST/Bayes	? typical spend only	table 3	both sexes engage similarly riskily, although males have higher typical month spend. The lack of relationship with last monthly spend possibly due to seasonal/smoothing effects
H1.1.8b	age	loot box engagement (RLI/spend)	positive association	NHST/Bayes	? RLI only	table 3	specific link with risky loot box engagement; lack of association with expenditure due to lower earnings of young people
H1.1.8c	unemployment	loot box engagement (RLI/spend)	positive association	NHST/Bayes	−	table 3/electronic supplementary material, table S1	sample size related?
*H1.2*	*(b) correlations weaker with non-randomized (i.e. not loot box) game-related purchases*						*(c)*
H1.2a	problem gambling (PGSI)	game spend	association larger with LB than nLB cohort	Bayes CI	+	section ‘H1.2a–d’	
H1.2b	problem gaming (IGD)	game spend	association larger with LB than nLB cohort	Bayes CI	−	section ‘H1.2a–d’	problem gamers at risk of other types of predatory monetization—not just loot boxes
H1.2c	gambling cognitions (GRCS)	game spend	association larger with LB than nLB cohort	Bayes CI	−	section ‘H1.2a–d’	a difference, albeit non-significant
H1.2d	impulsivity (BISB)	game spend	association larger with LB than nLB cohort	Bayes CI	−	section ‘H1.2a–d’	impulsivity linked specifically with risky loot box engagement (see H1.1.6 above), and not game spend
*H2.1–H2.6*	*(d) hypotheses about putative financial/psychological harms*						
H2.1	income	loot box engagement (RLI/spend)	no association	NHST/Bayes	? RLI and typical spend only	table 3	hypotheses largely supported, albeit small, significant correlation with last monthly spend
H2.2a	problem gambling (PGSI)	loot box expenditure	association greater when normalized to earnings	Bayes CI	−	table 5	earnings unrelated to loot box harms
H2.2b	problem gaming (IGD)	loot box expenditure	association greater when normalized to earnings	Bayes CI	−	table 5	earnings unrelated to loot box harms
H2.2c	wellbeing (WEMWBS)	loot box expenditure	association greater when normalized to earnings	Bayes CI	−	table 5	earnings unrelated to loot box harms
H2.3	wellbeing (WEMWBS)	loot box engagement (RLI/spend)	inverse correlation	NHST/Bayes	? RLI only	table 3	specific link with risky loot box engagement
H2.4	distress (K-10)	loot box engagement (RLI/spend)	positive correlation	NHST/Bayes	? RLI only	table 3	specific link with risky loot box engagement
H2.5a	loot box motivations: Distraction/Compulsion sub-scale	wellbeing	inverse correlation	Bayes CI	+	section ‘H2.5a–b’	
H2.5b	loot box motivations: Distraction/Compulsion sub-scale	distress	positive correlation	Bayes CI	+	section ‘H2.5a–b’	
H2.6	problem gambling problem gaming (PGSI/IGD)	loot box expenditure	independent contributions	multiple regression	+	section ‘H2.6’	

*H1.1–H.1.1.8: (A) bivariate correlations with psychological instruments and demographic variables*

For our pre-registered hypotheses about bivariate correlations between loot box engagement and various psychological and demographic variables, we predicted that the associations would be robust for all measures of loot box engagement (i.e. the RLI, our primary outcome; mean monthly spend; last monthly spend); and that results would also be confirmed by both frequentist and Bayesian statistical tests. The majority of these hypotheses were supported. See table 2 for a summary of results, table 3 for full results of bivariate correlations and table 4 for results with the RAFFLE scale. We summarize each result, in turn, below.

**Table 3.** Results of bivariate correlations. The first two columns list the predictor variables and the related hypotheses; where hypotheses are predicted to be a significant relationship between the predictor variable and outcome variables (RLI; loot box (LB) typical month spend; LB last month spend). The four sets of three columns are grouped into results; with the first set of three columns related to loot box outcomes in the LB cohort (RLI; LB typical month spend; LB last month spend) followed by a final set of columns related to non-random purchasing (the nLB cohort). For the final set of results, for non-random purchasing, no data are available for the RLI or the RAFFLE (as these instruments are specifically designed for loot box purchasing). Each set of four columns displays effect sizes for Kendall's tau, FDR adjusted *p*-value and Bayes factors. For the categorical variables of sex and employment status, there are no effect sizes reported (see Methods). Results are colour coded, where green = significant results across all tests (FDR adjusted *p*-value < 0.05; Bayes factors > 3); red = non-significant results across all tests; yellow = inconsistent results.

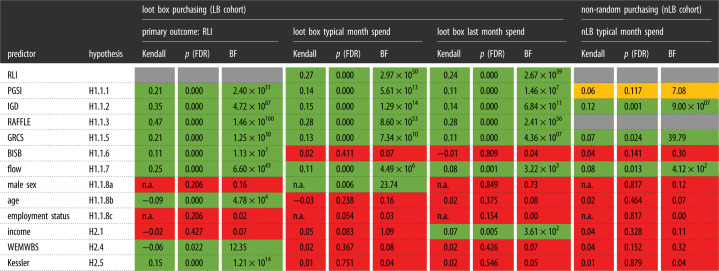

**Table 4.** Effects size estimates between RAFFLE sub-scales and measures of loot box engagement. The first column lists the RAFFLE sub-scales. The first set of results (left panel) is versus the RLI; second panel is versus loot box monthly spend. Each panel of results provides the estimate of effect sizes (Kendall's tau b) with 95% Bayesian credible intervals. The final column of each panel ‘95% intervals no overlap’ lists whether 95% credible intervals for each sub-scale overlap with the Distraction/Compulsion sub-scale (i.e. thereby establishing that the Distraction/Compulsion sub-scale has a significantly stronger association with RLI or loot box spend).

RAFFLE sub-scale	RLI				LB spend			
	estimate	95% CI		95% intervals	estimate	95% CI		95% intervals
		LL	UL	no overlap		LL	UL	no overlap
Distraction/Compulsion	0.45	0.42	0.48	n.a.	0.21	0.18	0.24	n.a.
Enhancement	0.35	0.32	0.38	Y	0.24	0.20	0.27	N
Progression	0.17	0.14	0.2	Y	0.08	0.04	0.11	Y
Social	0.30	0.27	0.34	Y	0.14	0.11	0.17	Y
Altruism	0.08	0.04	0.11	Y	0.09	0.05	0.12	Y
FOMO	0.32	0.29	0.35	Y	0.19	0.16	0.23	N
Resale	0.24	0.21	0.27	Y	0.19	0.16	0.22	N

*H1.1.1: problem gambling*

Results established a positive relationship between problem gambling symptom scores, measured by the PGSI and all measures of loot box engagement (table 3).

*H1.1.2: problem gaming*

Results established a positive relationship between problem gaming symptom scores, measured by the IGD and all measures of loot box engagement (table 3).

*H1.1.3: loot box motivations*

Results established a positive relationship between total score for loot box motivations, measured by the RAFFLE and all measures of loot box engagement (table 3).

*H1.1.4: loot box motivations: Distraction/Compulsion sub-scale*

In our pre-registration document, we predicted that the Distraction/Compulsion sub-scale of the RAFFLE would be most strongly linked with loot box engagement. The results establish (table 4) that for our primary outcome, the RLI, Distraction/Compulsion had a stronger association (i.e. larger effect size with Kendall's tau) than the other sub-scales. Furthermore, this difference between effect sizes was established as significantly different by inspection of 95% Bayesian CIs, which do not overlap between the Distraction/Compulsion and other sub-scales. With our secondary outcome (typical monthly loot box spend) the results were more equivocal. While the Distraction/Compulsion sub-scale again had a stronger relationship (Kendall's tau effect size) with spend than other sub-scales, the effect was not significantly larger than the Enhancement, FOMO and Resale sub-scales. Overall, however, the results do support our predicted hypothesis: the Distraction/Compulsion sub-scale is the strongest predictor of risky/problematic loot box engagement.

*H1.1.5: gambling cognitions*

Results established a positive relationship between gambling cognitions, measured by the GRCS and all measures of loot box engagement (table 3).

*H1.1.6: impulsivity*

Results established a positive relationship between impulsivity, measured by the BISB and ‘risky’ loot box engagement (measured by the RLI; table 3). There was no relationship, however, between impulsivity and other measures of loot box engagement (typical/last monthly spend). This suggests that impulsivity is specifically linked with risky loot box engagement.

*H1.1.7: experiences of flow*

Results established a positive relationship between experiences of game-related ‘flow’, as measured by a five-item set of the Game Experience Questionnaire, and all measures of loot box engagement (table 3).

*H1.1.8a, b, c: sex, age, employment*

In our pre-registration document, we predicted that loot box engagement would be associated with male sex, younger age and lower employment status. Only some of these hypotheses were supported (table 3). There was no association of male sex with RLI (our primary outcome) or last monthly spend, but there was an association with typical monthly spend (see Discussion). While younger age was associated with risky loot box engagement, there was no association with typical/last monthly loot box spend. There was no significant relationship between employment and any measure of loot box engagement; although from all the categories of employment status, unemployed had the highest mean typical monthly spend compared with other categories (see electronic supplementary material, table S1, for full data; where unemployed = £30.8; mean of all other employment statuses = £20.3).

*H1.2a–d: (B) correlations weaker with non-randomized (i.e. not loot box) game-related purchases*

In our pre-registration document, we predicted that for problem gambling, problem gaming, gambling cognitions and impulsivity, correlations with measures of engagement would be stronger for loot boxes than for non-randomized content. These hypotheses were tested by inspection of 95% Bayesian CIs. The results indicate that the problem gambling, as measured by the PGSI, indeed had a stronger relationship with purchasing loot box versus non-loot box related content (PGSI: LB cohort 95% CI 0.11–0.18; nLB cohort 95% CI 0.01–0.08). However, our hypotheses were not supported with measures of either problem gaming (IGSD: LB cohort 95% CI 0.11–0.18; nLB cohort 95% CI 0.06–0.14), impulsivity (BIS-Brief: LB cohort 95% CI −0.01–0.05; nLB cohort 95% CI 0.00–0.08) or gambling cognitions (GRCS: LB cohort 95% CI 0.09–0.16; nLB cohort 95% CI 0.03–0.10).

*H2.1–H2.6: (C) hypotheses about putative financial/psychological harms*

Our final set of hypotheses were focused on the putative psychological and financial harm of loot box engagement, where we predicted that loot box engagement would be associated with higher psychological distress and lower wellbeing, and that associations with problem gambling and gaming would be higher when loot box expenditure was normalized to earnings (due to the higher financial burden of loot boxes on lower earners).

*H2.1: no association between loot box engagement and income*

By contrast to associations between loot box engagement and constructs such as problem gambling and gaming, we hypothesized that income *would not* be associated with loot box engagement. While there was no association between income (BF < 0.3) and RLI (our primary outcome), and no evidence of an association with typical loot box monthly spend (inconclusive BF), there was an association between income and last monthly spend (tables 2 and 3).

*H2.2a–c: association with problem gambling, gaming and wellbeing would be stronger when loot box spend is recalculated as a proportion of self-reported income*

We predicted that associations between loot box expenditure and problem gambling, gaming and wellbeing would be stronger when loot box spend is recalculated as a proportion of self-reported income. These hypotheses were tested by manual inspection of 95% Bayesian CIs (table 5), where results did not support any of the hypotheses, and established no difference between effect sizes (i.e. all CIs were overlapping).

**Table 5.** Associations of loot box spend versus normalized looot box spend. A number of our hypotheses (H2.2a–c) proposed that various associations would be stronger when loot box spend was normalized to income (i.e. as a proportion of income) when compared to non-normalized spend. These hypotheses are listed in the second column, relating to PGSI, IGD and WEMWBS having stronger associations with normalized income. The following two panels of results provide the estimate of effect sizes (Kendall's tau b) with 95% Bayesian credible intervals (associations with loot box spend on left panel; associations with loot box spend, normalized to earnings on right panel). The final column ‘95% intervals no overlap’ lists whether 95% credible intervals for loot box spend versus normalized spend overlap (i.e. thereby establishing there is a difference between correlations of loot box spend versus normalized spend).

instrument	hypothesis	loot box spend			loot box spend, normalized to earnings			
		estimate	95% CI		estimate	95% CI		95% intervals
			LL	UL		LL	UL	no overlap
RLI		0.27	0.23	0.3	0.23	0.19	0.26	N
PGSI	H2.2a	0.14	0.11	0.18	0.13	0.09	0.16	N
IGD	H2.2b	0.15	0.11	0.18	0.12	0.09	0.16	N
RAFFLE		0.28	0.24	0.31	0.26	0.22	0.29	N
GRCS		0.13	0.09	0.16	0.08	0.05	0.12	N
BISB		0.02	−0.01	0.05	0.08	0.04	0.11	N
flow		0.11	0.07	0.14	0.08	0.05	0.12	N
age		−0.03	−0.07	0	−0.13	−0.16	−0.1	Y
WEMWBS	H2.2c	0.02	−0.01	0.06	−0.03	−0.06	−0.01	?
Kessler		0.01	−0.03	0.04	0.07	0.04	0.11	?

*H2.3: higher loot box engagement (RLI) will be inversely correlated with wellbeing, as measured by the ‘Warwick–Edinburgh Mental Well-being Scale (WEMWBS)*

We predicted that higher loot box engagement would be inversely correlated with wellbeing, as measured by the WEMWBS. Results were mixed (table 3) establishing an inverse relationship between wellbeing and our primary outcome, the RLI (albeit of small effect size), but no significant relationship with spend.

*H2.4: higher loot box engagement will be positively correlated with psychological distress, as measured by the Kessler Psychological Distress Scale (K-10)*

We predicted that higher loot box engagement would be positively correlated with psychological distress, as measured by the K-10. Results were similar to wellbeing, establishing the predicted relationship with the RLI, but no association with loot box spend.

*H2.5a–b: higher scores on the Distraction/Compulsion sub-scale of the RAFFLE will be inversely correlated with wellbeing and positively correlated with distress*

We predicted that scores on the Distraction/Compulsion sub-scale of the RAFFLE would be inversely correlated with wellbeing (WEMWBS; H2.5a) and positively correlated with distress (K-10; H2.5b). Results support both hypotheses, where the Distraction/Compulsion sub-scale of the RAFFLE has a reverse correlation with wellbeing (Kendall's tau = −0.10; *p* < 0.001) and a positive relationship with distress (Kendall's tau = 0.20; *p* < 0.001).

*H2.6: independent contributions of problem gambling and gaming to loot box engagement*

Finally, we predicted that problem gaming and problem gambling scores would independently contribute to loot box engagement, i.e. such that participants who have both higher problem gambling (PGSI) and higher internet gaming disorder (IGD) scores will spend significantly more on loot boxes than participants with higher scores on just one of these measures. As pre-specified, a linear regression was conducted to test for an interaction between PGSI and IGD as predictors of typical loot box expenditure. The results were as expected, with a non-significant interaction between PGSI/IGD (*t* = 0.07, *p* = 0.207). An equivalent regression was also conducted with RLI as the outcome, also showing no interaction between PGSI and IGD (*t* = −0.00, *p* = 0.204).

## 4. Discussion

### 4.1. Discussion of pre-registered hypotheses: (a) bivariate correlations with psychological instruments and demographic variables

#### 4.1.1. Psychological variables

With psychological instruments (i.e. for gambling; gaming; loot box motivations; gambling cognitions; impulsivity; flow), the results generally supported hypothesized associations (6/7 supported; 1/7, for impulsivity, supported only against our primary outcome, the RLI; table 2). Such results confirm previously well-established associations with both problem gambling and problem video gaming [6,10]. Furthermore, these two variables appear to independently contribute to loot box expenditure (see H2.6). Additionally, our observed association with gambling cognitions supports the findings of a single previous survey [29], thereby adding to growing evidence that loot boxes share many psychological characteristics with traditional gambling.

Impulsivity was associated with risky loot box engagement (our primary outcome variable), but not loot box expenditure. This may elucidate the equivocal evidence for links between impulsivity and loot box engagement in the existing literature [17,18], where expenditure rather than risky involvement was measured. Moreover, a recent publication [27] also found RLI scores to be more strongly associated with impulsivity than expenditure; although several sub-scales of impulsivity were weakly (but nonetheless significantly) correlated with loot box spend. Accumulating evidence, therefore, suggests that impulsivity is linked with risky loot box engagement [29].

Our study is the first to establish links between loot box engagement and experiences of game-related ‘flow’. A passive version of the flow experience, labelled ‘dark flow’, has been previously linked with problematic gaming [42], where it might offer an escape motivation from mental health issues such as depression [40,41]. It is possible that an analogous situation might happen with loot boxes.

Our results with the RAFFLE scale may shed further light on such a hypothesis: while observed relationships between the RAFFLE and loot box engagement are unsurprising (i.e. because the RAFFLE is an aggregate measure of motivations for loot box purchasing), our results did establish that the Distraction/Compulsion sub-scale is the most strongly linked with risky loot box engagement. Similar to gambling, facilitation of a flow-type state might be one mechanism whereby loot boxes offer ‘distraction’ from daily life or feelings of distress. This could happen in two ways: the flow offered by the gameplay itself (where loot box purchases might be required to continue satisfactory gameplay), or alternatively a ‘dark flow’ type state induced by the act itself of opening the loot boxes. Here, some interview participants in our previous study reported feeling compelled to purchase loot boxes in certain games to obtain items required to get past a ‘pinch point’ and continue playing [11]. Further investigations are required to fully understand how game-related experiences of flow might mediate escape motivation—especially from mental health issues—and how this could act as an inadvertent driver of risky loot box engagement.

#### 4.1.2. Demographic variables

Results were generally unsupported regarding our hypotheses around bivariate correlations with demographic variables (male sex, age, employment status).

While typical self-report monthly spend was associated with male sex, last monthly spend was not. This may be explained by a combination of seasonal effects (i.e. with games like FIFA, with updates released alongside the start of the new football season) in addition to the ‘smoothing’ properties of self-reporting the ‘typical’ monthly spend (i.e. ‘typical’ monthly spend will be less variable than last monthly spend). These smoothing properties are detectable in the lower variance in typical monthly spend compared to recent monthly spend (s.d. 71.94 versus 62.11). Moreover, males had larger variance in spend than females; especially for last monthly spend (typical monthly spend, male mean = 27.5, s.d. = 83.78 and female mean = 16.30, s.d. = 30; last monthly spend, male mean = 20.34, s.d. = 102.16 and female mean = 11.92, s.d. = 18.46). In other words, there is substantially more month-to-month volatility in the loot box expenditure of males than females—possibly relating to seasonal effect with gendered games such as FIFA. This may explain why typical monthly spend differed between the sexes, whereas last monthly spend did not—and our data collection did not overlap with the September start of the football/FIFA season. Future research is required to investigate the possibility of such sex-related seasonal effect. An alternative explanation—and perhaps complementary one—relates to recall bias, where the sexes are known to differ with respect recall bias [63,64], and where recent monthly spend should suffer less from recall bias.

Whatever the reasons for this contradiction, the results here do not contradict our previous findings [22] or the work of others [65], which have reported differences between the sexes with regard to loot box expenditure. First, these previous studies have tended to use the longer timeframes [65]—and our results establish such differences between males and females in typical monthly spend. Second, our study (as far as we know) is the first to investigate the relationship between sex and risky loot box engagement— establishing no differences between the sexes. In other words, while males might spend more on loot boxes, they do not appear to engage with them more riskily—an observation that is consistent with spend data being subject to differences in recall bias between the sexes. Confirmatory research is required.

With younger age, while we observed positive correlations with RLI (our primary outcome), we did not observe correlations with loot box expenditure. A likely explanation is that while younger age is linked with ‘risky’ or problematic loot box engagement, the lower earnings of younger people means that these results are not observed with loot box expenditure (i.e. due to the higher disposable income of older people; where age and income are significantly positively correlated in our cohort, Kendall's tau = 0.32, *p* < 0.001). Again, such a result is corroborated by our previous published results [22]: while younger age was associated with the binary decision to engage with loot boxes, it was *not* associated with loot box expenditure—*except* after normalization of expenditure to earnings. In other words, while younger gamers do not spend more on loot boxes, they do spend *a higher proportion of their income and engage with them more riskily*.

Furthermore, this result (i.e. a specific relationship with risky loot box engagement) mirrors our observed results with impulsivity—where younger people are known to be more impulsive [66] implying that impulsivity and younger age might specifically associated with problematic/risky loot box engagement.

Finally, for employment status, we observed no differences between any measure of loot box engagement and employment status. This is contrary to our earlier, much larger survey [22]. One possible explanation relates to sample sizes: in the current survey, we again observed higher typical monthly spend for unemployed versus other statuses (£30.8 versus aggregate of £20.3 for all other employment status; see electronic supplementary material, table S1, for fully disaggregated results), although the results did not reach significance across the various six different categories of employment status in the ANOVA test. This suggests that such effects are only detectable in large, population-level survey data.

### 4.2. Discussion of pre-registered hypotheses: (b) correlations weaker with non-randomized (i.e. not loot box) game-related purchases

Results established that problem gambling symptom score was more strongly associated with loot box spending than with non-loot box game-related purchases. This result thereby reiterates narratives around the gambling-like structure and shared psychology of loot boxes and gambling [5], and that problematic gamblers may be at risk of additional harms from loot boxes.

By contrast, the results did not establish that problem video gaming, gambling cognitions or impulsivity were more strongly associated with either type of purchase. With problem video gaming, effect sizes were somewhat similar for both loot boxes and non-loot box related purchases; and with gambling cognitions, while effect sizes were somewhat different (0.13 versus 0.07 for typical monthly spend on loot boxes versus non-loot boxes), confidence intervals for these did overlap. These findings reiterate arguments around potential risks from other types of predatory monetization (i.e. beyond just loot boxes), especially for those who have difficulties controlling their gaming or with erroneous gambling-related cognitions [2].

For impulsivity, there was no association with expenditure on loot boxes *or* non-loot boxes—instead, we observed (hypothesis H1.1.6) that impulsivity was specifically associated with risky loot box engagement. This supports the idea that spend alone is not a fully comprehensive measure of impulsive/risky engagement with loot boxes, and that the pattern of engagement and the individual's self-reported feelings of (lack of) control over their purchasing decisions is an important indicator. Although impulsivity may be similarly linked with risky/impulsive purchases of other game-related content, the RLI is a specific instrument for loot boxes, and we, therefore, did not measure such impulsive purchases of other game-related content. Further research is required to investigate whether impulsivity is linked specifically with loot boxes, or is similarly associated with other types of game-related purchases.

Furthermore, while a number of previous studies have established equivocal results [17,18,25,26] for links between impulsivity and loot box engagement (albeit consistently small effect sizes, even for positive findings), more recent work investigating impulsivity as a multidimensional construct has established positive relationships between loot box engagement and sensation seeking, reward sensitivity and positive urgency [27].

### 4.3. Discussion of pre-registered hypotheses: (c) hypotheses about putative financial/psychological harms

While income was not associated with risky loot box expenditure or typical monthly spend, it was associated with last monthly spend (table 3). However, the effect size for this correlation was relatively small (0.07), meaning that the results broadly support our hypothesis that earnings are not a major driver of loot box engagement.

With player wellbeing (H2.3; that wellbeing would be negatively correlated with loot box engagement), our results were mixed (table 3). While we did not observe any association with expenditure, we did observe a negative relationship with risky loot box engagement. Similar results were observed with distress (H2.4)—i.e. a specific link between distress and risky loot box engagement, but not general expenditure. Such results suggest that distress and negative wellbeing may be specifically linked with risky loot box engagement. While this finding at first seems contradictory, it is consistent with previous results in the literature [37], where loot box spending has been associated with both positive and negative moods, albeit with small effect sizes.

Due to existing evidence that higher-spending players are disproportionately more likely to be problem gamers and gamblers, but do not have higher earnings [22], we predicted that associations between loot box expenditure and problem gambling, gaming and wellbeing would be stronger when loot box spend is recalculated as a proportion of self-reported income (table 5). The results, however, did not reveal any differences between these correlations depending on whether loot box expenditure was normalized or not. Such results instead establish that earnings do not have a major influence on relationships between loot box expenditure and problem gambling/gaming. Interestingly, the relationship with wellbeing was *negative* after loot box spend was normalized to earnings, possibly due to the higher wellbeing of higher earners. However, distress *was* more strongly associated with loot box expenditure when normalized to earnings; again, broadly consistent with observations that loot box expenditure may be linked with both positive and negative moods [37].

Finally, when specifically investigating motivations of loot box purchase linked with ‘Distraction/Compulsion’, we observed an inverse correlation with wellbeing and a positive relationship with distress. This sub-scale contains items encompassing escapism which is associated with disordered gaming [31] and gambling [32], and items encompassing ‘urge’, which is also strongly associated with problematic gambling. These results suggest that putative harms of loot boxes may be linked with specific types of motivations, and may relate to earlier observed results with game-related experiences of ‘flow’.

### 4.4. Limitations and future work

Our sample was limited to a convenience sample of UK adults from the Prolific platform, which may be liable to similar selection biases to other online surveys [67], although there is evidence that it surpasses both MTurk and undergraduate student samples on a number of data quality measures [68,69]. Nonetheless, our aim was to use this online platform to purposively sample gamers that make game-related purchases. Future work should involve both children and demographically representative cohorts from other nations. Furthermore, while sociodemographic categories on Prolific are convenient, they fall short of standards for national monitoring, especially around ethnic categorization [70]. Similar to earlier work, our survey uses self-report measures of loot box expenditure, where such self-report approaches are liable to poor reliability when compared to more objective or direct measurement [71]. While self-report approaches for gambling expenditure have previously been endorsed as generally reliable [72], subsequent evidence has shown participants make estimation errors for both gaming and gambling [73–76]; similar investigation of loot box expenditure is warranted. However, self-report spend data are widely used in research, including our own, because of the challenges in obtaining account data. Furthermore, previous studies have highlighted that links between gambling and loot box engagement may be driven by respondents confusing loot boxes as comprising genuine gambling [56]—and while we did make efforts to defend against such confusion within our survey wording, further research is needed into such potential confounders.

While our results with flow are novel, our study pragmatically used the same five-item scale that had been previously deployed in studies on gambling [40] and videogaming [77]. Given the complexities of an experience such as ‘flow’, more basic and theoretical research (such as qualitative studies) is required, where five items are unlikely to capture the full breadth and nuance of experience. There is also debate about flow, including its nature as a psychological construct, how to measure it and the brain mechanisms that might give rise to it [78–80]. A new scale made available after we completed data collection shows potential in capturing some of these complexities [81], but does not discriminate between ‘positive’ and ‘dark’ flow. There may be scope for future scale development work. Again, similar to other work, our analysis did not pay attention to different types or genres of games, where specific game types (e.g. free to play; mobile games, etc.) may be played more by specific cohorts, thus acting as a further potential confounder.

Also similar to earlier work, our survey uses cross-sectional approaches: any directions of causality between various behaviours cannot be robustly established, although we have previously found preliminary evidence for loot boxes acting as a gateway into other forms of gambling for a subset of gamers, and recent longitudinal studies have established migration from loot boxes to gambling in adolescent [82] and young adult cohorts [83]. Nonetheless, even if gamblers are simply more heavily engaged with loot boxes, a risk of harm still exists [17]. Further longitudinal research, experimental approaches and qualitative work are required to explore gamer's perceptions of the complex, multidirectional relationships between these various overlapping psychological variables and behaviours. Finally, our results emphasize the utility of measures such as the RLI and the RAFFLE, which routinely exhibit stronger associations with psychological variables and are less likely to suffer from various recall and other biases associated with spend data [84].

## 5. Conclusion

Our survey establishes associations between loot box engagement and variables including problem gambling, problem gaming, impulsivity, gambling cognitions, experiences of flow and specific links with motivations of ‘Distraction/Compulsion’. Links between videogame purchasing and problem gambling (but not problem video gaming) were larger with loot box purchasers than non-loot box purchasers, reiterating the gambling-like nature (and risks) of loot boxes. Nonetheless, such results also highlight that some individuals (e.g. problem gamers) may also be at risk from other potential types of non-loot box ‘predatory monetization’ [16].

Concerning the potential psychological harms of loot boxes, we observed links between our primary outcome variable, risky loot box engagement, and both distress and negative wellbeing. Similarly, we also observed significant relationships between ‘distraction and compulsion’ motivations and both distress and negative wellbeing.

Overall, the pattern of these various correlations hints at potentially fruitful areas for future work, especially around relationships between impulsivity and game-related experiences of flow to potentially drive compulsive and risky purchasing behaviour, with subsequent impacts on wellbeing and distress. Whatever the underlying nature of these relationships, it is clear that these various psychological variables are inter-related in a complex manner. Therefore, to gain insight into the nature of these relationships—including which variables are most predictive of loot box engagement—we pre-specified several Bayesian mixed-effects regressions. These are discussed in a second, linked publication: ‘The psychology of loot box engagement, part 2: exploratory analyses of complex relationships’*.* Unravelling the influences of various psychological and demographic influences on loot box engagement, and subsequent financial and psychological harms, would help support the targeting of future interventions, such as education, age and spending limits and regulatory enforcement.

Whatever the underlying nature of these relationships, our results reiterate the shared gambling-like characteristics of loot boxes, and that specific cohorts may be at risk of enhanced harms, thus reiterating ongoing calls for loot box legislation for harm-minimization purposes. With the UK government adopting an approach to loot boxes of industry self-regulation [85], it is known that industry compliance to features such as odds disclosures and game labelling (through statements such as ‘In-Game Purchases Includes Random Items') are consistently unsatisfactory [86–88]. Some online storefronts even have compliance rate as low as only 7.1% [87]. Moreover, such measures have doubtful utility [89]: they are poorly comprehended and largely ignored by adults, children and parents alike. Unless ‘tangible results begin to be seen in the near future’ (as requested by the UK’s Department for Digital, Culture, Media and Sport [85]), then they should ‘not hesitate to consider legislative options…to protect children, young people and adults'. Such legislation should include a range of consumer protection measures, including enforced age restrictions and customizable spending limits, acting alongside mandatory, clear and upfront labelling and odds disclosures [2].

## Data Availability

Both works have been pre-registered on OSF (https://osf.io/a5nwj), and all underlying data and analysis code are available also (https://osf.io/gh634). Supplementary material is available online [90].
